# Passive UHF RFID Tag for Multispectral Assessment

**DOI:** 10.3390/s16071085

**Published:** 2016-07-14

**Authors:** Pablo Escobedo, Miguel A. Carvajal, Luis F. Capitán-Vallvey, José Fernández-Salmerón, Antonio Martínez-Olmos, Alberto J. Palma

**Affiliations:** 1 ECsens, CITIC-UGR, Department of Electronic and Computer Technology, ETSIIT University of Granada, Granada E-18071, Spain; pabloescobedo@ugr.es (P.E.); carvajal@ugr.es (M.A.C.); amartinez@ugr.es (A.M.-O.); 2 ECsens, Department of Analytical Chemistry, Faculty of Sciences, University of Granada, Granada E-18071, Spain; lcapitan@ugr.es; 3 Institute for Nanoelectronics, Technical University of Munich, Munich DE-80333, Germany; jfsalmeron@nano.ei.tum.de

**Keywords:** screen printing, printed electronics, passive RFID tag, optical sensor, microcontroller, spectral fingerprint

## Abstract

This work presents the design, fabrication, and characterization of a passive printed radiofrequency identification tag in the ultra-high-frequency band with multiple optical sensing capabilities. This tag includes five photodiodes to cover a wide spectral range from near-infrared to visible and ultraviolet spectral regions. The tag antenna and circuit connections have been screen-printed on a flexible polymeric substrate. An ultra-low-power microcontroller-based switch has been included to measure the five magnitudes issuing from the optical sensors, providing a spectral fingerprint of the incident electromagnetic radiation from ultraviolet to infrared, without requiring energy from a battery. The normalization procedure has been designed applying illuminants, and the entire system was tested by measuring cards from a colour chart and sensing fruit ripening.

## 1. Introduction

In its simplest form, a radio frequency identification (RFID) tag is composed of an antenna and an integrated circuit (IC). Most popular RFID tags are low-cost passive tags that draw energy from the electromagnetic field radiating from the reader when they are within range of its interrogation zone [1]. In this regard, printed electronics are a good solution to manufacture thin, wearable, flexible, lightweight, and cost-effective electronic devices [2]. There are several examples of RFID tags produced with printing techniques such as screen printing, gravure, flexography, and inkjet printing, among others [3,4,5,6,7].

There is growing interest in including sensing capabilities in RFID tags in order to expand their features, adopting different solutions to achieve this aim. On one hand, some authors have associated the tag’s analog response (read range, resonance frequency, backscattered power, etc.) with a variation in the monitored magnitude [4,8,9,10,11,12,13,14]. However, although quite valuable results have been achieved, several factors could interfere in link performance, making it difficult to distinguish whether the change in the tag’s analog response is in fact due to a variation in magnitude or to some other interference. Moreover, the RFID reader generally needs to include additional circuitry to detect a tag response change. On the other hand, there are some other approaches to design the sensor tag based on passive, semi-passive or active operation modes proposed for packaging surveillance applications [15,16]. Some of these designs combine microcontroller architectures with RFID chips to incorporate different environmental sensors together: temperature, light, and acceleration [17]; temperature, humidity, and gases [18,19,20]; ion or gas concentration [21,22,23]; pressure [24,25,26]; temperature with surface acoustic wave (SAW) sensors [27,28]; or general RFID platforms [29,30,31,32,33]. Other single-chip sensing architectures without a microcontroller unit have also been reported [34,35,36]. These strategies have the advantage of both processing the sensor data directly in the RFID tag and reducing costs. In addition, chipless RFID tags do not require any IC in the transponder as they encode the data in the frequency or the time domain [37,38]. This chipless approach has been used to develop tags sensing temperature [39], humidity [40,41], surface cracks [42], gases [43,44], and visible radiation [45].

In this work, we present the design, fabrication, characterization, and validation of a passive printed RFID tag with multiple optical sensing capabilities. The main novelty is the ability to measure up to five magnitudes from different sensors with a unique, fully passive printed tag. The tag, directly powered from the electromagnetic field that the RFID reader radiates, includes five photodiodes to cover a wide spectral range: infrared (IR), visible region (blue (B), green (G), and red ((R) components), and ultraviolet (UV). These sensors provide useful information to detect spectral data, and their practical applications can be extended to areas such as colourimetry and optical chemical sensing [46]. The tag has been printed on a flexible substrate using screen-printing technology, making it adaptable to different shapes and useful in smart packaging applications. The tag architecture boosts the sensing capabilities of the RFID chip, allowing the use of five external sensors with a single chip, as explained in Section 2. In Section 3, two possible applications are tested, both in the context of reflective colour sensing. The first application consists of a simple colour checker of plain-coloured cardboards. In the second application, the tag is used for identifying the stage of banana ripening, from immature to overripe.

## 2. Materials and Methods

### 2.1. Sensor Tag Architecture

**Figure 1:**
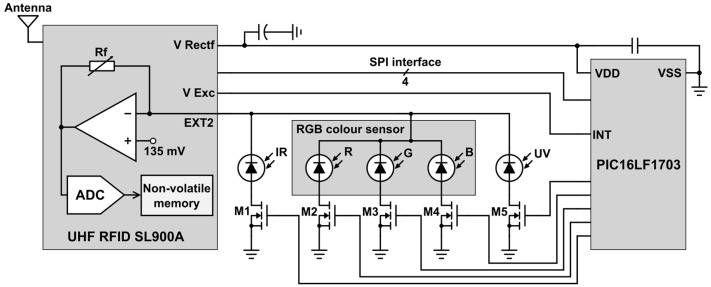
Schematic diagram showing the optical sensor mode of the RFID chip and its connection with the five photodiodes and the microcontroller unit.

Figure 1 shows a schematic diagram of the RFID tag architecture. This architecture was presented in rigid FR4 substrate in a previous conference paper [47]. The tag comprises an SL900A RFID chip (AMS AG, Unterpremstaetten, Austria) compatible with the EPC Gen 2 RFID standard [48], a microcontroller unit (MCU) PIC16LF1703 (Microchip Technology Inc., Chandler, AZ, USA), an RGB colour sensor KPS-5130PD7C (Kingbright Electronic Co., Issum, Germany) with three silicon photodiodes (one each for red, green, and blue), a Silicon PIN IR photodiode TEMD7100X01 (Vishay Intertechnology, Inc., Malvern, PA, USA), a UV Schottky-type photodiode GUVA-S12SD (Roithner LaserTechnik GmbH, Vienna, Austria), and extra circuitry to interface the optical sensors.

Moreover, the RFID chip has a fully integrated temperature sensor and two inputs for external sensors. We used this RFID chip in a previous work where two sensors were handled with no MCU [24]. Now, we have included a MCU to expand the possibilities of sensor reading with a novel strategy based on controlling a low power array of analog switches as it will be explained below. The tag has a passive architecture, in which the antenna harvests the energy necessary for powering up the system from the reader’s EM field. The antenna was designed to resonate at 868 MHz, corresponding to the UHF European RFID band. The radio frequency interface includes an RF Surface Mount Device (SMD) inductor to match the chip input impedance [48].

The RFID chip is directly connected to the microcontroller, which was selected for its low power consumption (nanoWatt XLP technology). The microcontroller communicates with the RFID chip by means of a Serial Peripheral Interface (SPI) bus. The RGB colour sensor and the IR and UV photodiodes are connected to the Sensor Front End (SFE) of the RFID chip, which comprises different sensor conditioning stages and a 10-bit Analog to Digital Converter (ADC). The voltage input range of the integrated ADC is set with two voltage references (V_o1_ and V_ref_) selectable in 50 mV steps from 160 mV to 610 mV. In addition, the V_o1_ voltage reference can be directly tied to ground. The V_ref_ voltage defines the lower voltage limit, while the upper voltage limit is defined by 2V_ref_ − V_o1_. These voltage references allow the selection of a particular resolution and range. As shown in Figure 1, the sensor conditioning stage internally consists of an operational amplifier with negative feedback, combined with a selectable feedback resistor R_f_ (185, 400, 875, 1875, or 3875 kΩ). The non-inverting input is fixed at 135 mV. Therefore, this configuration is a current-to-voltage conversion stage with selectable gain by choosing the value of the feedback resistor. It allows measurements with an optical sensor based on a reverse-bias diode current, such as a photodiode. The input voltage in the ADC V_ad_ is related to the reverse diode current:

I_D_ = (V_ad_ − 135 mV)/R_f_ (1)

**Figure 2:**
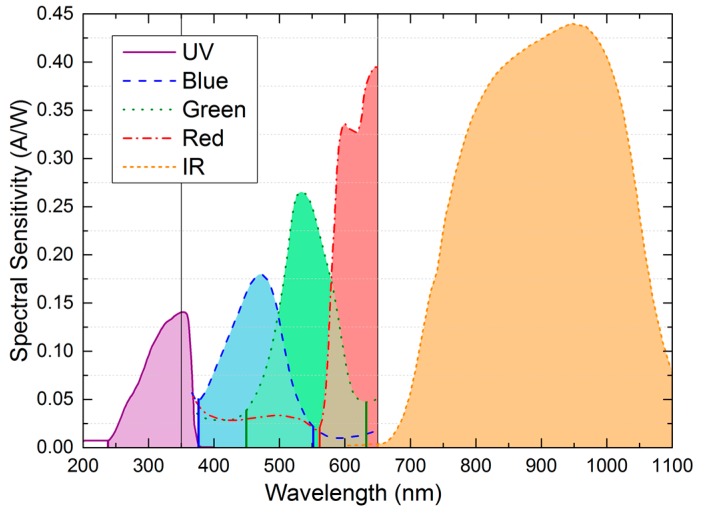
Spectral sensitivities of each photodiode according to manufacturer’s data. Vertical lines at 350 nm and 650 nm delimit the available manufacturer’s data for the RGB colour sensor.

Our RGB colour sensor is composed of a three-channel Si photodiode sensitive to red (peak sensitivity wavelength λ_p_ = 620 nm), green (λ_p_ = 550 nm), and blue (λ_p_ = 470 nm) spectral regions. The TEMD7100X01 infrared photodiode has a peak sensitivity at 950 nm and the GUVA-S12SD is sensitive to ultraviolet radiation (λ_p_ = 350 nm). Each photodiode channel generates a current proportional to the incident radiation. Figure 2 depicts the spectral sensitivity of each photodiode according to the manufacturer’s data [49,50,51].

In theory, the chip does not require any external components to read out a voltage value. However, since we wanted to sequentially measure the output of each photodiode in the same access, we included a switch based on five discrete MOS transistors combined with the microcontroller unit. The transistors are BSS138LT1 NMOS (Semiconductor Components Industries LLC, Phoenix, AZ, USA) with a resistance value in on-region R_DS(ON)_ = 3.5 Ω. Each transistor is connected to the anode of each photodiode (Figure 1). The sensors have a common cathode connected to the inverting input of the operational amplifier (EXT2 pin in the RFID chip). The gates of the five transistors are connected to different microcontroller outputs. The microcontroller generates the switch control signals, sequentially activating each output at a time. Therefore, one photodiode (ultraviolet, blue, green, red, or infrared) is read at each access. Every time an output is active, the microcontroller sends an SPI command to the RFID chip to measure the corresponding photodiode current. Then, the microcontroller saves the sensor readings using SPI commands on a specific location of the RFID chip’s non-volatile memory so it can later be accessed by the RFID reader. At this point, we must state that the proposed switch array to interface more than one sensor can be scaled up not only to five sensors, as presented here, but also to a higher amount of them taking into account the availability of MCU I/O ports that can be used to drive the switches. Therefore, we have designed a generalizable electronic architecture as interface between multiple sensors and a single RFID chip for the development of passive sensing tags.

**Figure 3:**
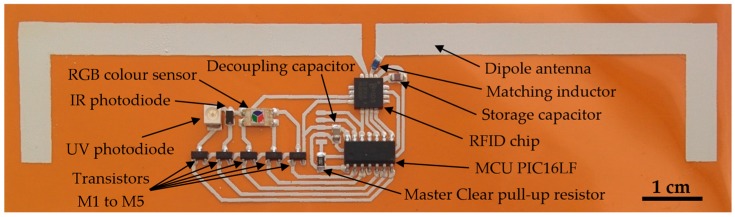
Screen-printed RFID tag on flexible substrate with all components.

In order to measure each photodiode output current, it is necessary to properly select the feedback resistor value and the ADC input voltage range. In the case of the feedback resistor, the RFID chip includes an autorange function that automatically selects a proper R_f_ value to provide unsaturated measurements within the ADC input voltage range. As stated above, the limits of the ADC input voltage are V_ref_ and 2V_ref_ − V_o1_. By default, these reference voltages are V_o1_ = 0 V and V_ref_ = 310 mV, so the ADC input voltage ranges from 310 mV to 620 mV, leading to a detection range of reverse diode current from 45.24 nA (with R_f_ = 3875 kΩ) to 2.62 μA (with R_f_ = 185 kΩ). Other detection ranges can be easily configured by choosing different values of V_o1_ and V_ref_. This is done through SPI commands sent from the microcontroller unit to the RFID chip. The minimum detectable current is 32.3 nA and the maximum one is 5.86 μA. Figure 3 shows the fabricated RFID tag with its main components labelled. A commercial RFID reader compatible with EPC Gen2, DK-UHF RFID HP2 (IDS Microchip AG, Wollerau, Switzerland) is used to power up and read the tag. The latter is done by sending EPC Gen 2 Read commands to access the RFID chip EEPROM memory locations where the measurements have been saved through SPI commands. The RFID reader antenna consists of a circular polarized patch antenna A0025 (Poynting antenna, Samrad, South Africa).

### 2.2. Fabrication Process

The tag pattern is directly printed on polyimide (PI) Kapton^®^ HN (DupontTM, Wilmington, DE, USA), a flexible polymeric substrate. The PI used has a thickness of 75 µm, a relative permittivity (ε_r_) of 3.5, and a loss tangent (tanδ) of 0.002. The screen-printing machine is a Serfix III (Seglevint SL, Barcelona, Spain). The screen used to fabricate the single-layer screen-printed tag has a mesh density of 120 nylon threads per centimetre (T/cm). The conductive silver-based ink is CRSN 2442 (Sun Chemical Corporation, Parsippany, NJ, USA). Under these conditions, the printed layer thickness is 13.3 µm and the resistivity is 39 ± 4 µΩ·cm [52]. After printing, the tag is cured at a constant temperature of 150 °C for 20 min to enable the formation of the conductive pattern and obtain the desired resistivity. To assemble the chips and the external components to the substrate, a three-step process was carried out. First, we interconnected the chips and silver pads by using the conductive resin H20E (Epoxy Technology, Inc., Billerica, MA, USA). After this, a double layer of 50-μm-thick dry adhesive, AR Clear 8932 (Adhesives Research, Inc., Glen Rock, PA, USA) was attached to the bottom of the chips to fix them to the substrate. Finally, a heating process was performed in an oven at 120 °C for 20 min to cure the conductive resin. The E5071C Vector Network Analyzer (VNA) (Keysight Tech., Santa Clara, CA, USA) was used to characterize the fabricated antenna. Considering the differential character of the antenna measurement, we defined the S-parameter differential port between port-1 and port-2 through a test fixture simulator included in the VNA. To connect the antenna to the VNA, we used Ultra Miniature Coaxial connectors (U.FL) (Hirose Electric, Tokyo, Japan) attached at the antenna feed point along with SMA to U.FL wires. For calibration purposes, a full custom Short-Open-Load-Thru (SOLT) calibration kit was used [35].

### 2.3. Methodology

To obtain the optical spectra as reference data in our experimental characterization and validation, we used a high-resolution spectrometer model HR2000+ (Ocean Optics Inc., Dunedin, FL, USA). This device has a spectral response range of 200 to 1100 nm and an optical resolution of 1.10 nm, with a 14-bit ADC module and a fiber light input method. The output of our RFID sensor tag consists of five current values (one for each photodiode) defining a spectral fingerprint. To adapt the spectral fingerprint obtained with our RFID tag, composed of five coordinates, to the spectrum measured by the spectrometer, the following procedure was carried out:

1. Standard normalization of the response of the five photodiodes according to their spectral sensitivities. The aim is to obtain an equivalent spectral graph with the same area under each photodiode response curve, but with a flat spectral sensitivity. To do that, we divide the area under the spectral sensitivity curve of each photodiode by its bandwidth (see Figure 2). The result of each division is the height of the flat sensitivity whose area is the same as the original spectral sensitivity. Then, we calculate the inverse of these heights. Finally, we normalize them to obtain the normalization factor assigned to each photodiode.
2. Multiplying each current value of the spectral fingerprint (obtained with our RFID tag) by each corresponding normalization factor calculated in Step 1. The result of this step is a set of five coordinates.
3. Assigning each of the five coordinates of the spectral fingerprint to the peak sensitivity wavelength of each photodiode (350, 470, 550, 620, and 950 nm).
4. Considering one of the values of the new spectral fingerprint and its associated wavelength. Then, calculating the factor necessary to get the same value as that obtained with the spectrometer at that particular wavelength.
5. Applying this calculated factor to the five coordinates of the spectral fingerprint.
6. Repeating Steps 4 and 5 for the rest of the coordinates.
7. The final spectral fingerprint is the average of all the values obtained for each photodiode. The error is calculated as the standard deviation of all the values for each photodiode from Steps 5 and 6.

This procedure was developed and optimized by comparing the spectral coordinates from our system to the spectrometer measurements for the studied illuminants. We used seven standard illuminants: white LED (cool and warm), infrared LED, UV lamp, fluorescent light, tungsten halogen lamp, and daylight. As a result, the calculated normalization factors for the five photodetectors were: 1.00 for the UV; 0.75, 0.57, and 0.30 for the blue, green, and red photodiodes, respectively; and 0.29 for the IR photodetector.

**Figure 4:**
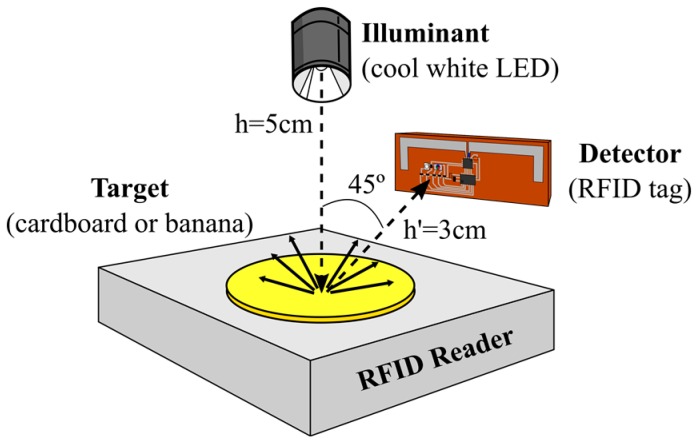
The 45°/0° geometry setup used to carry out diffuse reflectance measurements with the plain-coloured cardboards and the banana.

The next step was to validate this methodology by identifying cards from a colour chart. We carried out reflective colour-sensing measurements using several plain-coloured cardboards as targets: white, red, green, blue, orange, yellow, and brown. A cool white LED model XPGWHT-L1-0000-00H51 (Cree, Inc., Durham, NC, USA) was used as an illuminant. The geometric setup of the illuminant and detector consists of a typical 45°/0° geometry setup to carry out diffuse reflectance measurements (Figure 4). In this type of reflection, the target surface properties modify the spectrum of the incident light. The RFID reader is placed at the base of the setup to read the output of the photodiodes in the tag. The reader can be moved away from the target surface up to the maximum read range, which will be shown in the next section. To apply our system in a real situation, we then replaced the cardboards with a banana in order to monitor its stage of ripeness. Bananas change colour from green to yellow as they ripen, then becoming dark brown as they get overripe. The illuminant, setup, and procedure used in the banana experiment were the same as those used with the cardboards.

## 3. Results and Discussion

### 3.1. Antenna Characterization

**Figure 5:**
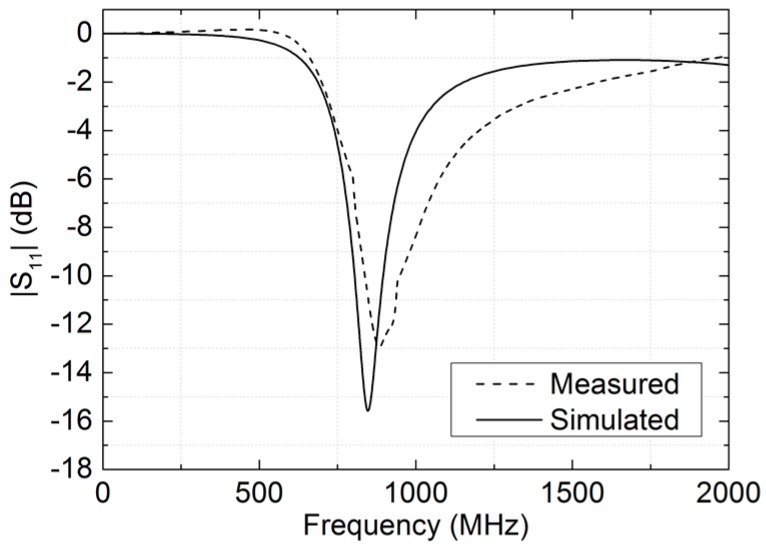
Simulated and measured differential S-parameter of the UHF dipole antenna showing minimal return loss at 868 MHz.

The antenna in Figure 3 is the typical dipole antenna based on the development kit recommended for the SL900A chip by the manufacturer (AG 2014), with final dimensions of the antenna arms of 5.5 mm in width and 79 mm in length. The dipole arms have been bent to optimize the occupied area. The input impedance of the dipole at 868 MHz is (31.1 + j9.4) Ω. This dipole is designed to achieve the same real part of the impedance as the RFID chip (31.1 − j286) Ω [53]. The imaginary part is compensated with an SMD matching inductor series 3650 of 51 nH and a quality factor of 60 at 900 MHz (TE Connectivity, Ltd., Schaffhausen, Switzerland) placed on one of its arms. Antenna gain is 0.661 dBi, directivity is 2.401 dBi, and efficiency is 66.97% at the working frequency. These parameters have been obtained by EM simulation with Advanced Design System 2013 (Keysight Technologies Inc., Santa Rosa, CA, USA). Figure 5 compares the dipole antenna measured response with the simulated one. The obtained resonance value at 868 MHz is −12.5 dB, whereas the simulated one is −13.6 dB. Additionally, we observed a wider range of response. The maximum read range can be calculated using a Friis free-space equation [54]:

(2) $${\text{range}}_{\text{max}}=\frac{\lambda }{4\pi }\sqrt{\frac{G_{\text{tag}}{\text{ G}}_{\text{reader}}{\text{ P}}_{\text{reader}}\;\tau \text{ PLF}}{S_{\text{tag}}}}$$

where λ is the wavelength, G_tag_ is the tag antenna gain, G_reader_ is the reader antenna gain, P_reader_ is the effective power transmitted by the reader, S_tag_ is the RFID chip sensitivity, τ is the power transmission coefficient, and PLF is the polarization loss factor. In our case, the transmission power is 26 dBm and the reader antenna gain is 7 dBi at 868 MHz, according to the manufacturer. The PLF includes the polarization mismatch between the reader antenna (circular polarization) and the tag antenna (linear polarization), with a value of 0.5 (−3 dB). The minimum threshold power necessary to activate the tag and answer the identification inquiries of the EPC protocol is S_tag_ = −15 dBm. However, extra power is required to drive the SFE [35] and power up the microcontroller unit. This power is collected from the reader’s radiated EM field. Therefore, the chip sensitivity to read out a value of the photodiode current is increased to −3.98 dBm. Assuming ideal conditions (τ = 1 and G_tag_ = 0.661 dBi as obtained by the EM simulation) and considering the chip sensitivity to be −3.98 dBm, the read range should be 1.5 m, according to Equation (2). The measured read range of 1.1 m for the RFID tag is slightly smaller than the simulated one, perhaps due to the non-ideal behavior of the printed conductive layer, reducing the tag antenna performance. To measure this range, the RFID reader antenna was attached to a tripod and placed in front of the tag at the same height. The measurements were taken in an anechoic chamber.

### 3.2. Tag Performance: Spectral Response

**Figure 6:**
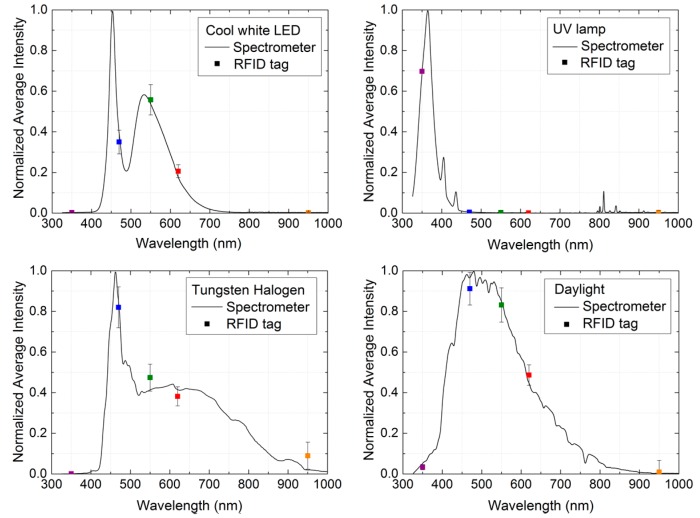
Comparison of the normalized spectral responses measured with the spectrometer and the spectral fingerprints obtained with the RFID tag for different types of illuminants: cool white LED, ultraviolet lamp, tungsten halogen light, and daylight. Lines are spectrophotometer outputs and dots are spectral fingerprint coordinates.

Figure 6 shows the final step of an iterative process where spectral fingerprint coordinates and the full spectral response are fitted. Error bars come from the standard deviations calculated in the final step of the methodology explained in Section 2.3. 

Four illuminants are placed directly facing the photodiodes in the tag for this figure. The resulting procedure for computing the spectral fingerprint is an estimation based on the spectral sensitivities provided by each photodiode manufacturer. Despite this, the normalized experimental results can be fitted to the spectral emissions of each illuminant. Therefore, after this optimization process and accounting for the normalization factor correction, the spectral fingerprint obtained allows the identification of the illuminant source applied to the tag.

### 3.3. Validation and Application in a Real Situation

**Figure 7:**
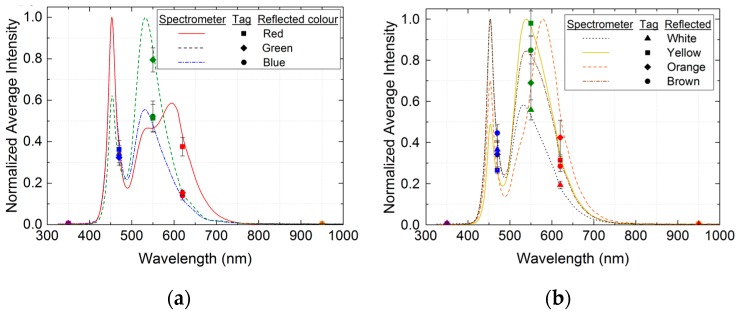
Comparison of the normalized spectral responses measured with the spectrometer and the spectral fingerprints obtained with the RFID tag for reflective colour-sensing experiments with: (**a**) red, green, and blue cardboards; (**b**) white, yellow, orange, and brown cardboards.

Apart from the possibility of identifying different illuminants, this passive tag has been used to conduct reflective colour-sensing measurements. We first carried out a validation of this type of measurement using a colour chart as the target. Figure 7 shows the results for seven plain-coloured cardboards: the three primary colours (red, blue, and green), white, yellow, orange, and brown. The illuminant is a cool white LED and the setup is as depicted in Figure 4. It can be observed that the spectrum of the incident light measured with the spectrometer has been modified in accordance with the cardboard surface colour. The spectral fingerprints obtained with the RFID tag also reflect the colour change, and the relative heights of its five coordinates allow the identification of the cardboard colour as well.

**Figure 8:**
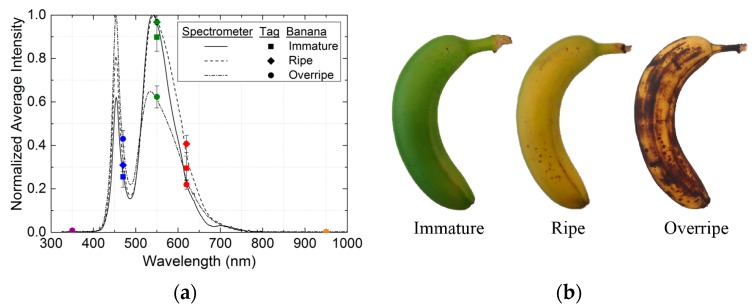
(**a**) Normalized spectral responses measured with the spectrometer and spectral fingerprints obtained with the RFID tag for the progress of banana ripeness from immature to overripe; (**b**) Photographs of the banana tested in its different ripening stages: immature, ripe, and overripe.

Along the same line, we propose an application for our RFID tag in a real situation: identification of the ripening stage of fruit. To this end, we consider a banana as the target and monitor the progress of its ripeness from immature to overripe (see Figure 8a). The banana can be classified as immature, ripe, or overripe according to the relative positions of the five components in its spectral fingerprint. Figure 8b shows photographs of the banana at each ripening stage. Given the colour shift from green to dark brown, an illuminant with a high green component, such as the one used in this study, could be the most suitable one to measure the ripening process.

Table 1 presents the spectral fingerprint values obtained with our RFID tag for the different targets considered: on one hand, the plain-coloured cardboards used for the validation process, and on the other hand, the banana used for its ripening stage identification. In these cases, most of the information is contained in the blue, green, and red coordinates. This is due to the spectral characteristics of the light source used as an illuminant. Nevertheless, this information is enough to identify the colour in the case of the cardboards and the ripening stage in the case of the banana. Other radiation sources with significant spectral information in the ultraviolet or infrared regions could also be used as illuminants.

## 4. Conclusions

This work describes a passive printed UHF RFID tag with multiple optical sensor capabilities. Five photodiodes have been integrated on the tag, covering a wide spectral range from ultraviolet to infrared regions. A microcontroller-based switch circuit has been designed and tested to allow the multiplexing of several photodiodes to one SFE input. Furthermore, the chosen RFID chip has a built-in temperature sensor. Therefore, six different magnitudes can be measured in every reading in a fully passive mode. The tag has been manufactured by screen-printing on a flexible polymeric substrate. This system boasts a very high sensing capability, in this case up to six different magnitudes in the same tag. The implemented switch array expands the capabilities of the sensor-enabled RFID chip, which includes at most two inputs for external sensors. As the switching process is sequential, this interface solution is scalable, the number of available MCU I/O pins being the only limitation to the number of sensors that can be interfaced.

The operation of this passive tag has been successfully demonstrated. First, using several standard illuminants, a procedure was defined to obtain the spectral fingerprint of the target illuminant. Therefore, these types of RFID tags would be very useful to detect different light conditions. Subsequently, tag performance was validated through reflective colour-sensing measurements. In this regard, the spectral fingerprints obtained with our RFID tag were modified by the target surface colour, using a cool white LED as an illuminant and different plain-coloured cardboards as targets. As the main limitation, we have detected some influence of the label/illuminant source position on the tag results that should be minimized in later refinements of this design. Also, the measured read range of the tag is limited to 1.1 m. Beyond this range there is no longer sufficient power to drive the SFE of the RFID chip and power up the MCU, whose measured power consumption in operation mode is 0.42 mW. Finally, we have proposed a real-life application using the same technique to identify the ripeness of a banana from immature to overripe. These results could be extended to the study of other fruits or food in general, and the tag could be used in smart packaging applications.

## Figures and Tables

**Table 1:** Spectral fingerprint coordinates obtained with the RFID tag for the reflective colour-sensing experiments with the plain-coloured cardboards and the banana ripening stages.

Target	Spectral Fingerprint Coordinates				
	UV	B	G	R	IR
White cardboard	0.007 ± 0.003	0.37 ± 0.04	0.56 ± 0.05	0.194 ± 0.018	0.0027 ± 0.0006
Red cardboard	0.006 ± 0.004	0.36 ± 0.04	0.51 ± 0.07	0.38 ± 0.05	0.0042 ± 0.0017
Green cardboard	0.005 ± 0.005	0.32 ± 0.03	0.80 ± 0.06	0.154 ± 0.011	0.0036 ± 0.0013
Blue cardboard	0.007 ± 0.003	0.34 ± 0.05	0.52 ± 0.08	0.140 ± 0.020	0.0018 ± 0.0007
Yellow cardboard	0.005 ± 0.005	0.265 ± 0.017	0.98 ± 0.06	0.314 ± 0.018	0.0051 ± 0.0014
Orange cardboard	0.005 ± 0.004	0.34 ± 0.07	0.69 ± 0.14	0.42 ± 0.08	0.0058 ± 0.0021
Brown cardboard	0.006 ± 0.004	0.45 ± 0.04	0.85 ± 0.07	0.29 ± 0.03	0.0050 ± 0.0023
Immature banana	0.0011 ± 0.0003	0.26 ± 0.05	0.90 ± 0.07	0.30 ± 0.06	0.00026 ± 0.00008
Ripe banana	0.00084 ± 0.00007	0.31 ± 0.03	0.968 ± 0.011	0.41 ± 0.04	0.00021 ± 0.00018
Overripe banana	0.008 ± 0.006	0.43 ± 0.04	0.62 ± 0.05	0.2198 ± 0.0021	0.0012 ± 0.0011

## Abbreviations

The following abbreviations are used in this manuscript:

UHF	Ultra high frequency
RFID	Radio frequency identification
IC	Integrated circuit
EM	Electromagnetic
UV	Ultraviolet
IR	Infrared
SMD	Surface mount device
XLP	Extreme low power
SPI	Serial peripheral interface
SFE	Sensor front end
ADC	Analog to digital converter
MOS	Metal-Oxide-Semiconductor
EEPROM	Electrically erasable programmable read-only memory
PI	Polyimide
VNA	Vector network analyzer
LED	Light emitting diode
PLF	Polarization loss factor
